# Part and Whole in Pictorial Relief

**DOI:** 10.1177/2041669515615713

**Published:** 2015-12-07

**Authors:** Jan Koenderink, Andrea van Doorn, Johan Wagemans

**Affiliations:** Laboratory of Experimental Psychology, Department of Brain & Cognition, University of Leuven (KU Leuven), Belgium; Faculteit Sociale Wetenschappen, Psychologische Functieleer, Universiteit Utrecht, The Netherlands; Faculteit Sociale Wetenschappen, Psychologische Functieleer, Universiteit Utrecht, The Netherlands; Laboratory of Experimental Psychology, Department of Brain & Cognition, University of Leuven (KU Leuven), Belgium

**Keywords:** Pictorial relief, shape segmentation, hills and dales, visual awareness, stereopsis

## Abstract

What are “natural parts” of pictorial reliefs? Intuitively, and suggested by common lore from the visual arts, they are the bulges that stick out toward the observer. Each such bulge contains a (locally) nearest point and is bounded by one or (usually) more curvilinear ruts. The latter meet in “passes” or saddle points. This divides the relief into “natural districts”. From a formal analysis one knows that reliefs can be divided into “hill districts” or “dale districts”, these two “natural” parcellations being fully distinct. We report empirical results that strongly suggest that visual awareness is based on a partition in bulges, which are mutually only weakly connected. Such a notion immediately explains why inverted reliefs or surfaces illuminated from below appear so different as to be mutually not recognizable.

## Introduction

This article addresses issues in “paradoxical monocular stereopsis”. It should not be confused with the contemporary definition of “stereopsis” which takes the causal effect of binocular disparity for granted.^1^ Stereopsis (Koenderink, van Doorn, & Wagemans, 2011) is simply the awareness of three-dimensional (3D) space. What the literature refers to as “paradoxical monocular stereopsis” (Claparède, 1904; Enright, 1991; Pollack, 1955; Koenderink, van Doorn, and Kappers, 1994) should perhaps be renamed “stereopsis in the absence of binocular disparity”, for that is simply what it is. It can also be experienced binocularly by offering both eyes identical optical input as happens with synopters, zograscopes, or stereoscopes loaded with identical pictures (Koenderink, Wijntjes, & van Doorn, 2013). There is nothing “paradoxical” about it, unless you are led to believe that depth is necessarily due to disparity. All visual artists and many nonscientists know that to be not the case (Ames, 1925a, 1925b; Schlosberg, 1941; Schwartz, 1971). Indeed, the term *paradoxical monocular stereopsis* is a fairly recent and very unfortunate one.

Henceforth, we use simply “stereopsis” for the sake of brevity (see Appendix A for a glossary of terms that we use in this article and that can be confusing to readers who do not know them or who are used to different meanings of these terms). Even for those who are singularly interested in binocular stereo, stereopsis proper should be relevant because the visual awareness experienced through binocular stimulation is at least partly, but probably largely, due to stereopsis proper (Koenderink, 2015). One reason is the low spatial acuity for disparity modulations (Rogers & Graham, 1982; Tyler, 1974), whereas the acuity for shading related relief is only limited by visual acuity. That is why binocular stereo presentations tend toward a “coulisses effect”, whereas stereopsis proper yields a “plastic effect” (Koenderink et al., 2013). With “plastic effect” we refer to the fact that pictorial objects usually appear as “rounded” (i.e., connected surfaces). With “coulisses effect” we refer to an arrangement of planes at different depths (e.g., foreground, middle ground, background), very much like cardboard cutouts or sections of theatre sets called “flats” or “wings” (e.g., Thompson & Bordwell, 2015). Appendix A contains some additional information on these notions.

Perhaps because conceived as “paradoxical”, monocular stereopsis has attracted only minor research efforts (Koenderink et al., 2011). This is a pity because it is evidently of key importance to vision in general. In the last two or three decades, we researched the topic extensively (Koenderink & van Doorn, 1995, 1998, 2003, 2012; Koenderink, van Doorn, Christou, & Lappin, 1996a, 1996b; Koenderink, van Doorn, & Kappers, 1992, 1994, 1996; Koenderink, van Doorn, Kappers, & Todd, 2001, 2004; Koenderink et al., 2011, 2012). Unfortunately, many issues stay unresolved. A major conceptual issue remains the way pictorial relief is mentally represented (Hildebrand, 1893). We can measure relief quantitatively on a point-by-point basis, and the results of such experiments have yielded very useful geometrical data (Koenderink et al., 1992). But although the measurements suggest that they might be due to sampling a coherent mental object, we have also found evidence to the contrary (Koenderink & van Doorn, 1995).

That there might exist something like a coherent mental representation is suggested by the fact that local measurements turn out to be globally coherent (Koenderink et al., 1992). This is a technical point that perhaps needs some explanation. Suppose you have a field of local samples of surface attitude, does that define a global surface? The formal answer is *no* (Koenderink, 1990). Most of such fields are not compatible with the existence of *any* surface (for an illustration and some discussion, see Appendix B). Surprisingly, the empirical answer is *yes*. Local measurements are apparently constrained by the mind such as to be compatible with a global surface, at least within the observational accuracy. This suggests the existence of some kind of data structure that forces such a constraint in the mind.

However, if this is indeed the case, then one expects observers to be able to use such a data structure. It turns out that observers cannot do this though. If they have to judge which of two points is closer, they can do so only if the points are on a single, uniform slope (Koenderink & van Doorn, 1995). They make mistakes when the points are on different hills or dales of the relief. This is remarkable because one could do better than the observer by using the data obtained in another experiment involving the same observer! So what is going on here? Perhaps the observer does not have access to mental data structures in all circumstances. A possible explanation is that the observer can only use the data structure piecewise. Possibly the data structure itself is not a whole, but rather a quilt of locally coherent, but mutually only weakly synchronized patches. Indeed, we have found indications for such a state of affairs (Koenderink et al., 2012).

In this study, we attempt to attack the local-global issue head-on by looking at nearer-farther judgements for points at arbitrary mutual separations, sprinkled uniformly over a pictorial relief.

## Methods

### Observers

In empirical studies on stereopsis, one has to face the problem that not all people experience it. Moreover, many people may not know that they actually do, but can be convinced of that in a few minutes through suitable instruction and demonstration, literally an “eye opener” (Schlosberg, 1941). When seeing a painting on the wall, one may look at the painting as a physical object (“generic mode”) or one may look into the painting and become aware of a pictorial space (“pictorial mode”). Phenomenologically, these awarenesses are quite distinct (Schlosberg, 1941), thus we will refer to them as different “modes” of vision. Appendix A contains some additional information on these notions.

It is probable that many varieties of stereopsis exist. Some people easily switch voluntarily between pictorial mode and generic mode, others can entertain both simultaneously, still others experience all pictures as planar objects (of course they are right about that!). This does not often surface in experiments because many (perhaps most) tasks can be done as well without stereopsis. A revealing difference is often response time, with perhaps fast responders relying on stereopsis, whereas others use cognitive strategies. However, this is not generally exploited in order to grade observers with respect to their visual repertoire.

Because of our research question we selected a small number of experienced observers who are known to experience stereopsis, the authors. Using a large number of naive observers would greatly complicate matters and force us to face numerous issues not immediately related to the key question. AD is female, aged 67, JK male, aged 72, and JW male, aged 51. They used their preferred correction. All had normal corrected acuity, use of binocular disparity, and trichromatic color vision (with JW being perhaps slightly deuteronomalous).

### Stimulus and the Geometrical Framework

**Figure 1.**
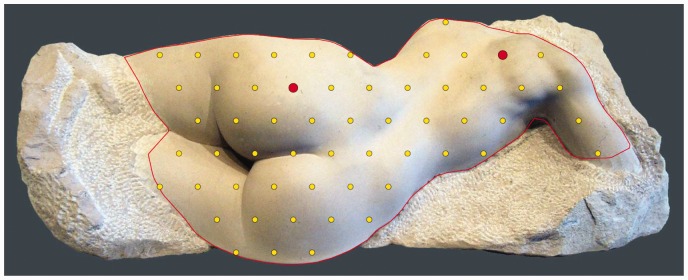
The stimulus and some geometrical framework. The picture is a photograph of a piece of sculpture (by Andrew Smith, see http://www.assculpture.co.uk/Figurative.html). The red contour defines our region of interest and yellow dots the vertices of a triangulation of its interior. The two red dots indicate a pair that might occur in a session. The task is to click the closest one with the cursor, using a mouse.

Figure 1 shows the stimulus and some important geometrical framework. We use only the region of interest indicated by the red contour. This area is triangulated, the yellow dots showing the vertices.

The triangulation is a rather coarse one because of various constraints. It counts 57 vertices, 133 edges, and 77 faces. With this number of vertices, one has 57(57−1)/2 = 1,596 orderless vertex pairs. They range in mutual separation from 0.056 to 0.661 times the width of the picture. The median separation is 0.224, the interquartile range (IQR) is 0.148 to 0.312.

These numbers are relevant because the task of the observers is to judge which member of a point pair appears to be closer to the observer.

### Presentation and Viewing

Observers viewed monocularly from a distance of 78 cm. They used a chin-rest to fix the vantage point.

The stimuli were presented on a DELL U2410f monitor, a 1920 × 1200 pixels (517 × 323 mm) liquid crystal display screen, in a darkened room. We used the standard Apple settings for white point and gamma. The stimulus filled the width of the screen. Above and below were empty black areas, except for a progress counter.

The fixed content was the picture, variable content were two marks. These marks were implemented as small pale-blue disks with a thin black outline. The marks were mutually identical, which is why we decided to omit reverse order presentations (Koenderink et al., 1996), thus saving on the number of pairs and thus gaining increased resolution.

### Sampling and Construction of the Pictorial Relief

The interface for the experiment is very simple. At each moment, the picture is displayed with two dots superimposed upon it. Observers had to click in the vicinity of the mark they perceived to be closest (i.e., a simple, intuitive task, which we have used successfully many times before, e.g., Koenderink & van Doorn, 1995; Koenderink et al., 1996; van Doorn, Koenderink, & Wagemans, 2011; Wagemans, Koenderink, & van Doorn, 2013). Although this can be done very quickly, the fact that there are 1,596 pairs renders this a time-consuming task (about an hour of intensely concentrated labor). The program simply selected the mark closest to the mouse location at click as the indicated point.

The resulting judgements are not necessarily mutually consistent, for one has 1,596 ordered pairs and only 57 vertices to order. One easily derives (van Doorn et al., 2011) that an optimal depth order is obtained by simply counting how many times a given vertex was considered closest. This yields a depth order to which the individual judgements may be compared. A number of merit is then defined exactly like Kendall’s tau (van Doorn et al., 2011). It is a useful check on the internal consistency of the observations. Thus, the basic analysis is very simple, due to the fact that we judge all pairs.

In this experiment, we are mainly interested in the *inconsistencies* because these reveal the nature of the mental representation (i.e., the pictorial relief; see Appendix A). In case the observer can simply “read out depth values” from a single globally coherent representation of the surface structure, for any location on the perceived surface, there should hardly be any inconsistencies and we would only derive trivial results from the data. The inconsistencies reveal the extent to which such a simple mechanism breaks down.

## Experiment 1

This is the main experiment. Each observer completed three sessions at different occasions. Observers experience the task as easy, and responses are fast. There is a dead-time of about a second, then responses take 0.5 to 2 s (IQR) with a median of 1 s. A session takes about an hour.

Observers resolve about 40 levels of the 57 and reach a number of merit of about 0.71 to 0.92. A typical number of discordant pairs is about 150. This is sufficient to derive interesting conclusions (see below).

The Kendal tau rank correlations between the depth orders from the three sessions are in the 0.71 to 0.93 range. The Kendal tau rank correlations between the depth orders from the mean results of the three participants are 0.58 for AD–JK, 0.54 for JK–JW, and 0.85 for JW–AD.

### Analysis

**Figure 2.**
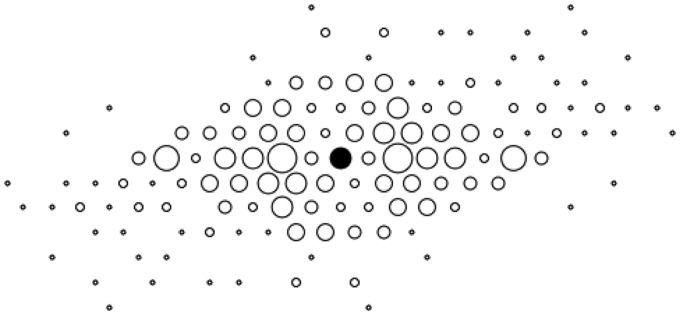
The number of confusions (proportional to dot diameter) as a function of the relative position of the point pair. Results are shown for all point pairs relative to the central point marked in black, for observer AD, session #1.

An analysis one might attempt is to consider the number of confusions as a function of the separation of the points. An example (for AD session no. 1) is shown in Figure 2. This representation reflects all pairwise measurements with the same vector length and orientation relative to the central point marked in black. Notice the central symmetry, which follows from this method. Results for other participants and sessions are very similar.

Although there seems to be a systematic pattern, this measure yields a distorted view through the fact that shorter separations are more numerous than longer ones. A regression of the probability of confusion against distance on the pooled data (all observers all sessions) reveals no significant dependence.

A more revealing approach is perhaps to attempt a cluster analysis using the probability of confusion as a distance metric (for different meanings of “distance”, see Appendix A). A simple definition for a suitable distance function is: *d*(*A*, *B*) = the distance *AB* when the points are not confused or the distance *AB* plus the diameter of the triangulation (the largest separation) when they are.

This metric is composed of two parts, namely the Euclidean distance in the picture plane, which is perhaps the default metric, and an all-or-none error metric (see Appendix A). Both are intuitively necessary, although it is not a priori evident how they should be blended. Our choice is perhaps the simplest one, and it turned out to work very well. We find that various alternative choices hardly make a difference except in extreme cases. Thus at least such a choice is not at all critical.

**Figure 3.**
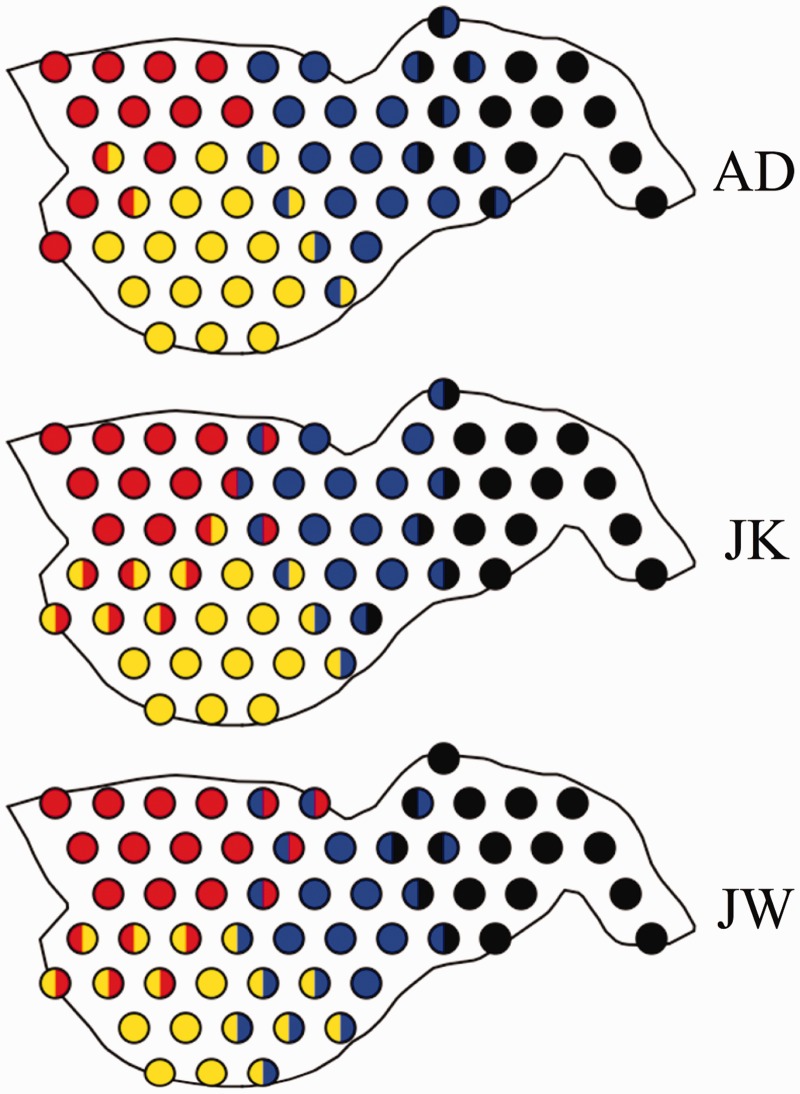
The clusters (indicated by different colors) for the three participants. These figures show the three sessions combined, thus some vertices on the boundaries occurred in more than a single cluster (multicolored in the figure). The fact that such cases are confined to boundaries indicates that the clustering procedure is rather robust.

**Figure 4.**
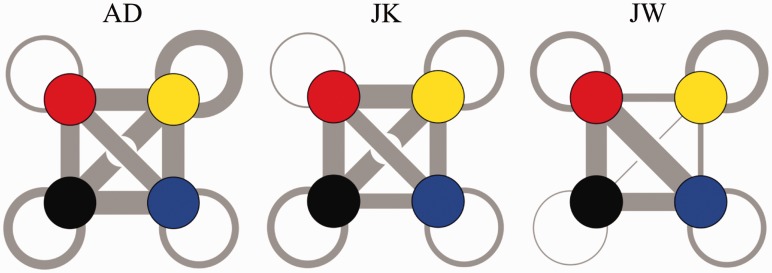
The thickness of the connections is taken proportional to the median of the number of confusions between two clusters over all sessions. Colors correspond to those used for the different clusters in Figure 3. Notice that confusions between clusters are far more likely than confusions within a single cluster. This indicates a certain degree of autonomy of the connected regions defined by the clusters. Some evident differences between the participants exist which are hard to spot in other representations.

With this error metric, a standard cluster algorithm^2^ converges to four clusters for all nine cases (three sessions for three participants). Moreover, these clusters are remarkably similar (Figure 3). We find that most of the confusions occur between, rather than inside clusters (Figure 4), thus showing that the distance function correctly discriminates. The regions belonging to the clusters turn out to be singly connected, thus showing that the clusters have geometrical significance. They indicate functional partitions of the area of interest.

The partitions defined by the clusters can be compared statistically through the Rand index (Rand, 1971). For the clusters obtained in the sessions for a single observer, we find values ranging between 0.84 and 0.89 (AD), 0.80 and 0.86 (JK), and 0.79 and 0.90 (JW). Two participants can be compared by finding the Rand index for all pairs of sessions. We find median values of 0.84 (IQR 0.83–0.88) for AD-JK, 0.86 (IQR 0.79–0.88) for AD-JW, and 0.84 (IQR 0.82–0.90) for JK-JW. We conclude that the partitions are very similar, as indeed visually obvious from Figure 3.

The pattern of confusions can be quantified by finding the ratio of the probability of an intercluster to an intracluster confusion. We find 2.04 for AD, 2.87 for JK, and 1.81 for JW. Thus, intercluster confusions are about twice as likely as intracluster confusions for all three participants.

## Experiment 2

This is an auxiliary experiment. We had observers sample local spatial surface attitude at the barycenters of the faces of the triangulation (Koenderink et al., 2001). As we have explained there, such observations allow one to find the depth relief sampled at the vertices. This “gauge figure method” is a well-understood technique that has been used in numerous applications. In essence, the observer adjusts an elliptical overlaid figure such as to “fit” the pictorial relief that is to say, to appear as a circle painted on the surface (Koenderink et al., 1992).

The auxiliary experiment is interesting because the gauge figure task is fully *local*. Will it fit the results from the 2-point comparisons, which are at least partly *global*? Of course, in the latter case, most comparisons involve points that are not too far apart, thus perhaps closer to “local” than “global”.^3^ So, the question asked here is this: Do such local surface attitude samples conform to the depth order from the 2-point comparison task?

Since the gauge figure task is a paradigm we have used many times, we do not discuss it in detail. Unfortunately, there are numerous ways to deploy this method in ways that ensure irrelevant results. Perhaps it is useful to mention the most common deviations from our paradigm here (for more discussion, see Koenderink & van Doorn, 2003):

- The gauge figure’s apparent spatial attitude *needs to be calibrated*. But against what? One answer would be against haptics, perhaps using a palm board. But what should calibrate what? Why suppose haptics and vision in isolation should necessarily agree? Which one is “right”? The very notion presupposes that perceptions ought to be “veridical” and thus invokes “God’s Eye” (Koenderink, 2014).
- The local samples need to be correlated somehow. A common solution is to show all gauge figures simultaneously and let observers iterate. But just consider what happens when one shows all gauge figures *simultaneously*. Then one may as well omit the picture entirely, the shape will be visible because of the sampling. This is evidently not a great idea.
- The haptic-visual interface fails to be natural. For instance, “Etch-the-sketch”-type implementations, which require the observer to use two knobs to set two directions at a single point, take unnecessary long time, and yield noisy results. Indeed, most adults are unable to write their own name with it at the first try, which is why it has become popular as a toy. Yet, we have seen many instances of such implementations.

### Analysis

We find that the rank correlations between the depth orders from the 2-point comparison task and the depths from the gauge figure task are substantial, namely (Kendall’s tau values) 0.734 for AD, 0.660 for JK, and 0.704 for JW. However, this is not to be considered *very* high. We often encounter much higher values in repeated sessions of the gauge figure method (e.g., see Koenderink et al., 2001). There are evidently differences that stand in need for further explanation.

**Figure 5.**
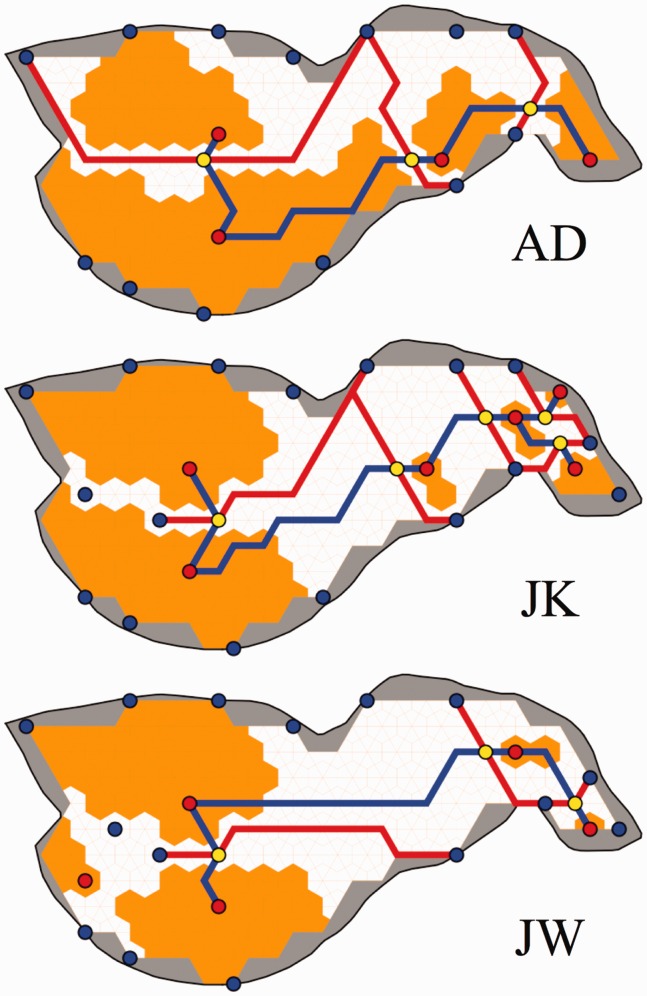
Geographical features for a fine-grained triangulation, mean depths over all (three) sessions obtained by the gauge figure method. Hill regions are shown as orange areas, ridges as blue lines, ruts (or water courses) as red lines, peaks as red dots, pits as blue dots, and saddles (or passes) as yellow dots. Notice that ridges and ruts pass through saddles and end at peaks and pits, respectively. Apart from these major features, there may be various minor subridges and subruts, but these tend to be different from session to session, whereas these major features are very robust. These features were computed from the averaged data per participant.

The gauge figure settings are very fast, thus it is easily possible to use a much finer grained triangulation. For such a fine-grained structure, we show the geographical features (hills, peaks, pits, passes, ridges, water courses (Cayley, 1859; Maxwell, 1870; Koenderink & van Doorn, 1998) as can be easily found by following the steepest descents into depth from all vertices (Figure 5). The geometrical foundation of this method is explained in more detail in Koenderink and van Doorn (1998) but the general idea is conveyed with an example in Appendix C.

Notice that the topology of the geography is very similar to the segmentation by the clusters discussed previously.

## Discussion

The cluster analysis probably yields the clearest representation of the results (Figure 3), especially when augmented by the graph structures indicating the bilocal^4^ nature of the inconsistent pairs (Figure 4). It is evident that pairwise depth comparison is better in certain subregions than it can be over the global relief. This is the case in spite of the fact that the region of interest suggests a smooth, connected relief.

Why is the region of interest broken up the way it is, in this case a segmentation into four subregions? We do not think that the number of subregions has a special meaning. For instance, it is probably unrelated to the magical number four as an estimate of memory capacity (proposed by Cowan, 2001), the subitizing range—the range of numbers that one can count in a single glance (e.g., Trick & Pylyshyn, 1994) or “FINST” for “FINgers of INSTantiation”—the capacity of visual attention or visual short-term memory as measured in multiple-object tracking (e.g., Pylyshyn & Storm, 1988). Any number larger than one would have enabled the same conclusion, namely that the pictorial relief is not entirely globally determined. On the other hand, it seems unlikely that one might find hundreds of these subregions in a study with our kind of resolution. There surely is some complexity bottleneck. If required to guess, we would put it almost certainly at less than 10. This is an issue that could be solved by (extensive) experimentation if one wished to find out (using techniques such as the one employed here), but it was not the focus of our study. Furthermore, the particular segmentation into four subregions is not likely to be due to chance because all three participants reveal essentially the same pattern. A possible answer may be found in the topographical structure of the relief. The pattern of hill-regions and the dissection of the area by ruts (or water courses) indeed suggest a basis for the segmentation.

**Figure 6.**
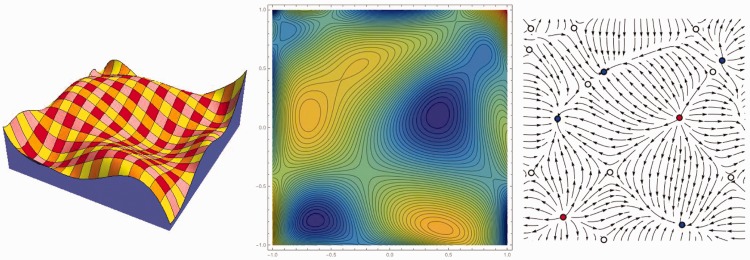
An example “landscape” with the geographical features as defined by Maxwell. At left a view of the relief, at center a map with equal-height (equally spaced) loci, at right a map with “streamlines”, that are the steepest descent courses taken by water running downhill. The summits are indicated as red, the immits as blue, and the passes as white dots.

The “geographical structure” of a relief is illustrated in the example (Figure 6). Suppose one desires to find the “natural hill districts”. Each summit defines such a district. It is found by running downhill from the summits into all available directions until one cannot follow the downhill course any further, which naturally occurs when one arrives at an immit. Different downhill courses may end up at different immits. The boundaries between these families of orbits are downhill courses that encounter a pass (or saddle) from which one has the choice of continuing toward either one of two distinct immits. Thus, the hill region is bounded by a curvilinear polygon whose vertices are immits, and on each of whose edges lies a pass. An analogous method serves to define “natural dale districts”, the only difference being that one has to move uphill instead of downhill. The edges of the hills are natural water courses (or ruts), whereas the edges of the dales are natural divides (or ridges).

If this is the correct interpretation, then it corroborates our speculative conclusion from above experiments, namely that observers have direct access to the depth variations over a single hill slope, but encounter difficulties when they need to compare a point on one slope with a second point on a different slope (Koenderink & van Doorn, 1995). Notice that both hill and dale districts are composed of “slopes”, and that a given slope is part of some hill and of some dale. Hills and dales are composed of distinct sets of slopes. One might speculate that the mental representation of relief is based on a segmentation of the region in terms of natural (hill or dale) districts (Cayley, 1859; Maxwell, 1870; Koenderink & van Doorn, 1998). The segmentation found from the 2-point depth comparison task suggests that the relevant “natural districts” are hills, rather than dales.

**Figure 7.**
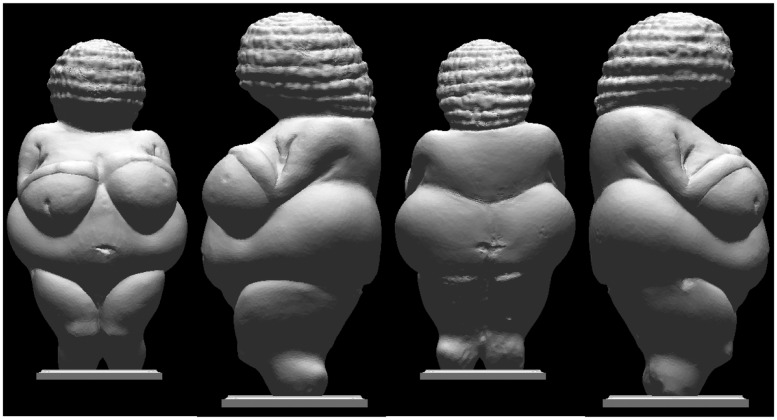
Rendering of the Venus of Willendorff from a number of directions. Illumination is frontal in all views. Notice that the statuette is designed as a conglomerate of ovoid shapes. As a result, one has a clear segmentation in terms of natural (hill) districts in the images. This greatly boosts stereopsis. It is even hard to see the images as what they “really are”, that are planar distributions of gray tones.

Such a notion fits well with art historical observations. For instance, the *Venus of Willendorff* (McDermott, 1996), a statuette dating from 28,000 to 25,000 BCE (the “Old Stone Age”) is divided into convexities by sharp ruts and poses an immediate, explicit segmentation of the view from any viewpoint (Figure 7). This fits in quite well with contemporary academic teaching of the art of sculpture (Rogers, 1969).

That vision prefers hills makes sense from the perspective of biology. Simple objects tend to be ovoid and to turn convexities toward the eye. Two convexities might well represent a pair instead of a single object. In such cases, it is the depth difference between the objects that counts, for that their individual reliefs are irrelevant. Suppose you see two “eggs” with one point indicated on each: What is the depth separation of the points? This question is similar to: How far is the Tour Eifel from the Brandenburger Tor? The answer is simply the distance Paris-Berlin. Their city plans are irrelevant to the question.

**Figure 8.**
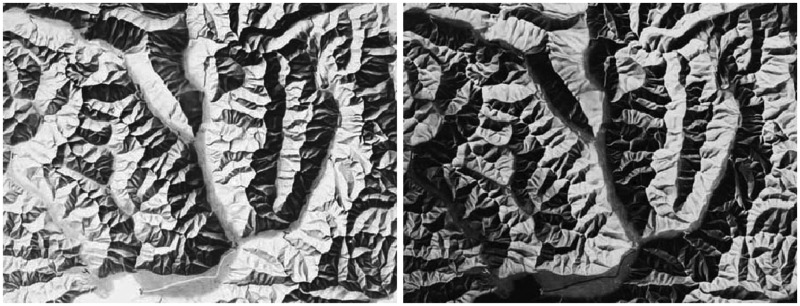
Example of the relief of an alpine landscape (left). At right, we show the image with the intensity scale inverted. In this negative the visually salient “parts” become very different.

**Figure 9.**
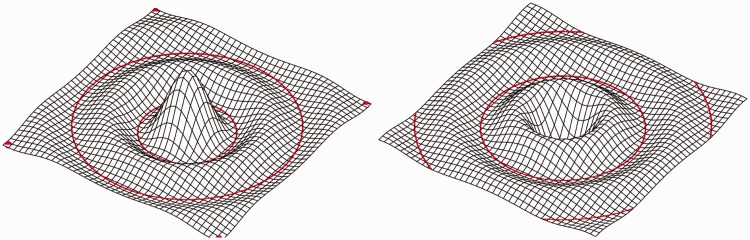
Two surfaces revealed through the deformations of a Cartesian coordinate mesh. The surface at right is the same as that at left except that we have inverted the vertical coordinate, thus swapping hills with dales. Notice that the visually salient “parts” become very different. (We have suggested the part-boundaries with the red curves.) Notice that this demo is similar to (but different from) that used by Hoffman and Richards (1984).

This also suggests a principled explanation of why reliefs appear so alien when their depths are inverted (Metzger, 1936; see Figure 8). In such a case hills become dales and vice versa, whereas natural dale districts are very different from natural hill districts. Thus *inverted reliefs have different parts* and as a consequence they elicit fully different global Gestalts (Hoffman & Richards, 1984; see Figure 9). Something similar happens when you illuminate an object from below: The dales turn into hills and the global impression cannot look normal—and, indeed, does not (Metzger, 1936)—because it has wholly unnatural parts.

## Conclusions

This study puts us in a position to answer the question posed at the introduction: Is the mental representation of pictorial relief a local or a global one? The answer appears to be that it is in-between. It is usually *not global*, with the exception of ovoid shapes. It is *not local* either, at least not in the sense of point-wise with some fixed size constraint. It is *piecewise* with the segmentation being similar to a distribution of “natural districts” (Maxwell, 1870). Notice that natural districts are either *hills* or *dales* and that vision singularly prefers hills. In extreme reductionistic cases concavities may be noticed, but a saddle shape (neither convex or concave),^5^ almost never is (van Doorn, Koenderink, Todd, & Wagemans, 2012; Wagemans, van Doorn, & Koenderink, 2010).

The present study affirms a tentative conclusion from a (much) earlier experiment (Koenderink & van Doorn, 1995). In that study, we also compared point pairs with respect to depth, but using a much finer triangulation. The advantage is resolution, the disadvantage the explosive increase in the number of pairs. The number grows with the square of the number of vertices, and the number of vertices itself increases inversely with the square of the triangulation’s edge length. Thus, we used only a few fiducial vertices and compared them with all others, thus forcing only a linear increase in the number of vertices. We were able to show that the observational scatter increased when points lie on different hill slopes as compared with a single slope. The former study was necessarily flawed by the fact that observers soon became familiar with the fiducial locations. The present study does not have this problem and has still a just sufficient resolution. In any case, our previous tentative conclusion was fully confirmed.

Of course, except from answering some questions, this study suggests many directions for follow-up studies. For instance, one could try to investigate the question about the number of clusters or regions alluded to above. Moreover, the clusters are very useful in suggesting interesting fiducial points, for instance their centers of gravity. This would enable a finer grained approach if one wanted to address the perceptual organization of pictorial relief at different spatial scales. Another potentially interesting topic is the segmentation induced by the clusters. This is a segmentation that is independent of various other ways of partitioning (e.g., like that illustrated in Figure 5). How do such partitions depend upon the image structure? This is an interesting question that can now be addressed with the methods developed here. In previous experiments, we have seen that human observers even segment the interiors of silhouettes or outlines (Koenderink et al., 2012). It seems to be an important strategy of the psychogenesis of visual awareness.

In sum, the part-whole structure of pictorial relief is very basic and suggests numerous novel explorations.

## Figures and Tables

**Figure A1.**
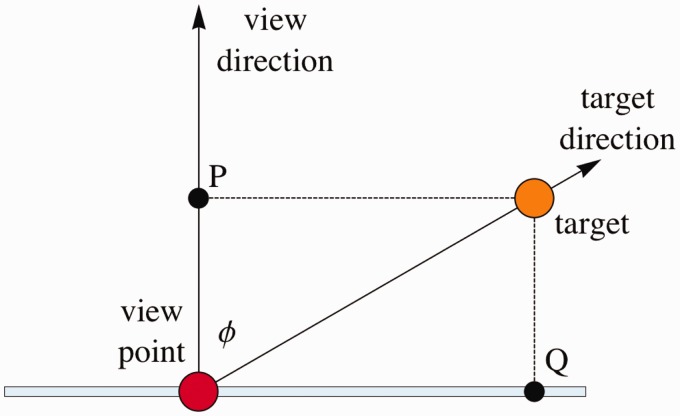
The distance from the viewpoint to the target is called “range”. The distance from target to Q (same as the distance from P to the viewpoint) is the more technical (optical) notion of distance.

**Figure A2.**
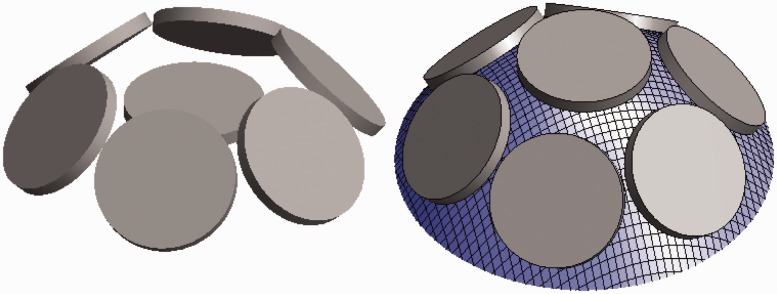
At left, seven empirically obtained samples of local surface attitude (slant and tilt) at a vertex of the triangulation and its six adjacent vertices. Here they are drawn “at the same depth”, but the measurements yield only surface attitudes, not depths. Assigning depth values in a coherent manner is not necessarily possible, but the case in this figure is an example where it succeeds. One can paste local surface patches together in a smooth manner; they neatly join up to form a convex “cap”. In this case, there is a smooth surface patch that “explains” (or interpolates) the local measurements; it is the blue paraboloid. This interpolating surface then lets us assign depth values—up to a common offset—to the vertices. This is how we calculate the depth relief on the basis of local surface attitude samples. Formally, one integrates a gradient field.

**Figure A3.**
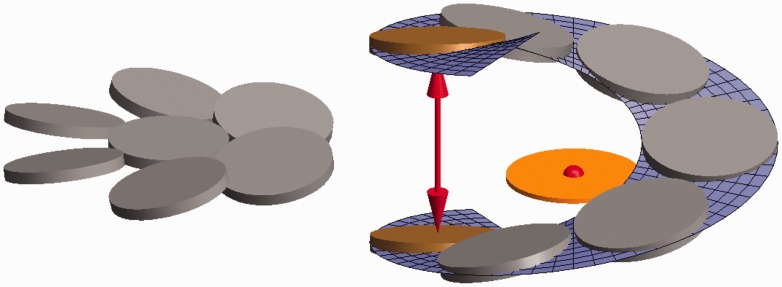
At left, another set of seven empirically obtained samples of local surface attitude (slant and tilt) at a vertex of the triangulation and its six adjacent vertices. Here, assigning depth values in a coherent manner is not possible. When one pastes local surface patches together in a smooth manner, the outer six samples form a staircase that fails to close; the red double arrow indicates the gap that cannot be bridged. Hence, in this case there is no smooth surface patch that “explains” the local measurements. Formally, the field of local surface attitudes is “non-integrable” because it is “not a gradient field”.

**Figure A4.**
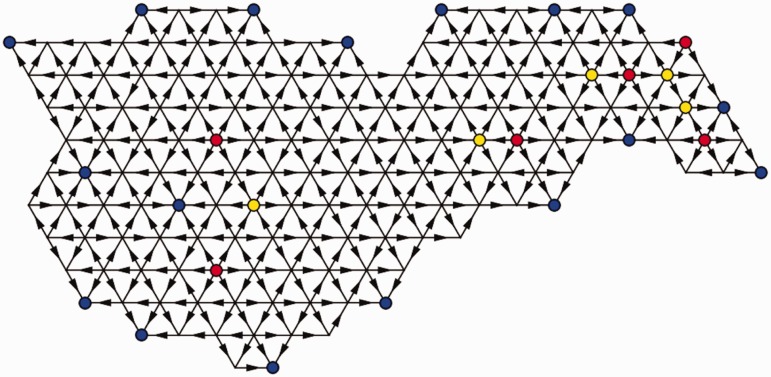
The triangulation edges with depths on the vertices define an oriented graph. We indicated peaks by red, pits by blue, and saddles by yellow dots. Notice that many of the pits are boundary minima. With some patience you may investigate hill regions by following the downhill stream. In practice such (in essence trivial) matters are best left to computer algorithms.

## Notes

1 For instance, www.oxforddictionaries.com has “The perception of depth produced by the reception in the brain of visual stimuli of both eyes in combination; binocular vision”. This is entirely typical.

2 We used Mathematica’s “FindClusters” algorithm, setting the method to “Optimize”, which is the default method.

3 Note that we use “local” and “global” as relative terms, with strictly local referring to a measurement in a single point on the surface and fully global referring to a measurement on the complete surface. Empirically, the degree of locality or globality can differ between experiments, depending on the nature of the measurement, the surface, the observer, and so forth. See also Appendix A.

4 We use “bilocal” to refer to relations between points on different hill regions (see also Appendix A).

5 The formal categories are “elliptic” (convex or concave) and “hyperbolic” (saddles), the taxonomy due to Gauss (1827).

## Appendix A. Glossary of Terms

### Stereopsis

In vision *stereopsis* is the awareness of a spatial quality, often denoted “depth” (i.e., a gradated sense of separation from the self; see point 2 below), that extends the spatial feelings usually denoted with “visual field”. The visual field is intuited as twofold extended, the two dimensions being generally similar in quality, though the vertical and horizontal are in some respects (as in the perception of bilateral symmetry, the distinction between squares and diamonds, etc.) phenomenologically distinct. The third dimension is not commensurable with the other two, even in the case of binocular stereopsis, for disparity fails to yield metrical range from the egocenter. Thus, visual space is similar to a “(2 + 1)-dimensional” space and not a 3D uniform and isotropic space. In monocular stereopsis, the third dimension is volatile and idiosyncratic although human observers are often able to reach common opinions on the spatial impressions evoked by generic photographic snapshots. The definitive modern source is the well-known book *Das Problem der Form* by the German sculptor Adolf von Hildebrand (1893). (Unfortunately, the English translation shows Hildebrand’s phenomenology through behaviorist eyes).

*Monocular and binocular stereopsis* are due to numerous cues and aspects of psychological set (“Einstellung”). Here we limit ourselves to monocular, static presentations, similar to looking at paintings from a fixed vantage point. It is common knowledge in the arts that such monocular stereopsis is not just possible but can be very compelling. This can also be traced in the literature of psychology (see our previous citation by Schlosberg). Monocular stereopsis is a fact that is important in various fields, for instance the arts. Hence, it seems most natural not to limit stereopsis to binocular stereopsis due to disparity. In fact, partly due to the low spatial resolution of the disparity cue, virtually all binocular presentations rely heavily on monocular cues (Koenderink, 2015). If “stereopsis” is mentioned one should indicate the viewing conditions (i.e., monocular, static as here vs. based on binocular disparity or motion parallax).

### Depth

“Depth” is a quale, essentially a feeling of separation from the ego which sometimes admits of quantitative differentiation. In the case of stereopsis, it is somewhat defined for various points in pictorial space. In practice it is operationally defined.

One operationalization is to have an observer estimate range. This is Fechnerian psychophysics and is often the intended meaning when talking about “depth perception” as opposed to “stereo”.

In many studies “depth” remains qualitative. This is phenomenology rather than Fechnerian psychophysics. Whitman Richards refers to “instant psychophysics” (Richards, 1987). Most of the literature on the “plastic effect” and so forth falls in this category. Our study (and most of our previous work on the topic) might be called “experimental phenomenology”. We operationalize something like depth (for instance, “pictorial relief”) in various ways.

In principle, all these “depths” are intrinsically different and their measure of correlation has to be addressed empirically.

### Coulisses Effect and Plastic Effect

These terms are common in the literature of art theory since (at least) the late 19th century. A core reference is Adolf von Hildebrand (1893) again. In this field, it is common knowledge that stereopsis occurs both monoscopically (static, no motion parallax) and through binocular disparity.

For instance: [When viewing a picture monocularly as opposed to binocularly] “the image opens up, as one says, that is to say, the water surfaces, roads, rows of columns run clearly in depth instead of from bottom to top, as they are painted; the illusion is much closer to volumetric reality than with two eyes open.” (our translation; Ebbinghaus, 1902, p. 424) and “… it should be stressed that the plastic depth that can be obtained monocularly is very striking, and must be seen to be appreciated” (Schlosberg, 1941, p. 601), whereas Schlosberg adds “A surprisingly large proportion of psychologists are unaware of the phenomenon, and somewhat at a loss for an explanation”.

The type of “*plastic depth*” (pictorial objects appear “rounded”) is often contrasted with the “*coulisses effect*” common in stereoscopy due to binocular disparity. Here is a recent citation from an account on 3D stereo movies: Most of the 3D films I’ve seen strike me as having two problems. First, there is the “coulisse effect.” Our ordinary visual world has not only planes (foreground, background, middle ground) but volumes: things have solidity and heft. But in a 3D film, as in those View-Master toys, or the old stereoscopes, the planes we see look like cardboard cutouts or the fake sections of theatre sets we call flats or wings (coulisses). They lack volume and seem to be two-dimensional planes stacked up and overlapping. (Thompson & Bordwell, 2015)

### “Pictorial” Versus “Generic” Mode of Vision

Certain observers report to have spatial experiences when looking into a painting, which looks very different to them when they merely look at it (like a flat object). We refer to the former impressions as being in the “pictorial mode”. It is not clear whether all or most observers sometimes experience this. When a painting is seen as a physical object, say a panel smeared with paint blotches, we refer to such experiences as “generic”.

### Pictorial Relief

“Pictorial relief” is used to indicate pictorial surfaces in pictorial space. Reliefs are smooth distributions of qualities like “depth”, “slant and tilt of surface elements”, “local surface shape”, and so forth. In the case of a depth field the “depths” are modulo an absolute depth and in the case of the spatial attitude of surface elements modulo an overall slant and tilt. Reliefs are intuitively surfaces of “pictorial objects”, the latter usually experienced as volumetric, although typically—that is in the case of opaque objects—the relief is where the vision “stops”, for neither the inside nor the backside of pictorial objects are “modally present”. For instance, a closed circular curve may be experienced as a hemispherical pictorial relief.

### Distance and Range

Distance is used in at least two different senses. The simplest is the familiar *Euclidean two-point distance*, which we consider generally understood. Then there is a *one-point distance*, usually simply called “distance”, which is the 2-point distance between a target and the viewpoint (the position of the eye in monocular viewing say). This second distance is only defined for points in a scene looked at by a monocular observer present in the scene. To complicate matters, this is not the distance used conventionally in technical optics (say photographic cameras) and linear perspective. In these cases, one does not only define a viewpoint but also a plane of projection (in linear perspective), focal plane (in photography), or frontoparallel plane (orthogonal to the primary visual direction of an observer). So we have a third definition of “distance”.

In Figure A1, the third distance is the length of the segment from Q to the target (same as the length of the segment from P to the viewpoint), whereas the second distance is the length of the segment from the target to the viewpoint. These “distances” are frequently confused in the literature. We propose to call the second distance “range”, the third (the technical convention) “distance”, where we have no immediate use for the first case.

### All-or-None Metrics

An all-or-none metric on a space simply assigns unit distance to two distinct elements and zero to the distance of any member to itself. It trivially satisfies the zero property, symmetry, and triangle inequality. Such metrics are of interest in the sets due to the partitioning by some equivalence relation. For instance, divisibility by two partitions the natural numbers into odd and even ones. An all-or-none metric might assign unit distance to an odd and an even number, distance zero to two even or to two odd numbers. Evidently this distance is nonnegative, and it is symmetric.

### Bilocal, or—More Generally—Multilocal Properties

A “local” property is defined at a single point. An example would be temperature. A slight complication is that one needs to decide on the size of a point. Since a temperature has to be based on numerous air molecules, the size has to be finite. In meteorology, one is happy with points the size of a mile. What is important is that a “point” is assumed to possess no internal structure. Remember Euclid’s definition “A point is that which has no parts”.

A direction can hardly be a local property, since a point—having no distinguishable parts—is an isotropic entity. A direction has to depend upon two distinct points, say A and B. The direction of the oriented stretch AB is evidently well defined. A “direction at a point” can be understood as the limit of AB, for B approaching A arbitrarily close. In the limit, one obtains what might be called a “bilocal property at location A”. This is similar to the velocity of a car; 60 mph does not mean one has to drive 60 miles in 1 hr, it is defined at any moment as any traffic cop knows.

## Appendix B. The Issue of Surface Coherency Obtained With the Gauge Figure Method

With the “gauge figure” method described in the article (first designed in 1992, since then used in many studies, both by ourselves and by others), we sample “surface attitude” at a large number of points on the picture surface. Surface attitude involves slant and tilt in depth of a local surface element. Formally one has dZ = f(X,Y) dX + g(X,Y) dY, where (X,Y) are Cartesian coordinates (say left-right and down-up dimensions) in the picture plane, and (dX,dY) small increments in the neighborhood of the location (X,Y). Then dZ is the depth increment encountered in the step from (X,Y) to (X + dX,Y + dY). Although Z indicates “depth”, only the step dZ is operationally defined. The method yields f(X,Y) and g(X,Y) at a number of locations (X,Y). The issue is: *Do such measurements imply a “relief”, that is a function Z(X,Y) up to some arbitrary constant?* The answer comes from elementary calculus: *Usually NOT, a necessary condition is* f_Y_−g_X_ = 0*, where* f_Y_ *stands for the partial derivative of* f *in the* Y*-direction* (*analogous for* g_X_, *etc*.).

To make this intuitive here are two examples. In the first example the condition is met, in the second it is not.

The first condition is simply f(X,Y) = X, g(X,Y) = Y, thus f_Y_ = 0 and g_X_ = 0, thus f_Y_−g_X_ = 0. Figure A2 shows a picture (left part) of seven surface attitude samples, six arranged symmetrically about a center point. The samples are presented at the same depths. In the right picture, we have shifted the samples individually in depth so as to “mesh”. They are now tangent to the smooth blue surface, which is the solution of the differential equation dZ = X dX + Y dY, that is Z(X,Y) = (X^2 ^+ Y^2^)/2 + constant.

In actuality there will be some “noise” due to measurement uncertainty, so one seeks a solution in the least squares sense, but this is merely a technical matter.

The second condition is simply f(X,Y) = Y, g(X,Y) = −X, thus f_Y_ = 1 and g_X_ = −1, thus f_Y_−g_X_ = 2. Thus, the condition is violated in the same measure everywhere. There is no integral surface possible. Figure A3 shows a picture (left part) of seven surface attitude samples, six arranged symmetrically about a center point. The samples are presented at the same depths. In the right picture we have shifted the samples—except for the center one (orange)—individually in depth so as to “mesh” as well as possible. They are now tangent to the smooth blue surface, which is like a smooth spiral staircase like one finds in many parking garages. One attitude sample is represented twice (brown), to show that there remains a gap that cannot be closed. This gap is indicated by the red double arrow. This problem occurs at any point, there is “no room” to force that ends meet.

In previous work (since 1992), we have shown that human observers yield attitude samples that allow a coherent surface fit (a “pictorial relief ”) up to deviations that are explained by the scatter encountered in repeated sessions. This is a highly remarkable result that suggests that there might be something like a representation Z(X,Y; up to an arbitrary constant) in visual awareness.

If this is indeed true, then one expects the observer to be able to decide whether Z(X_1_,Y_1_)−Z(X_2_,Y_2_) is positive or negative for any two locations (X_1_,Y_1_) and (X_2_,Y_2_). However, the present study shows that this is (in general) *not* the case. Observers can only do this if the locations coexist on a single hill. This is a finding of fundamental importance in the understanding of the structure of pictorial space.

## Appendix C. How to Derive the Geographical Features of the Pictorial Relief From the Gauge Figure Settings

The empirical results are depth values at the vertices of a triangulation. This means that the edges of a triangulation have a well-defined slope (equal depth values occur with probability zero). Thus, the triangulation can be represented as an oriented graph, the orientation of the edges denoting depth order. Figure A4 shows the result for observer JK, using the mean depth values over three sessions.

An interior vertex is incident with six edges. These can be divided into edges that leave the vertex and edges that enter the vertex. Typical edges have a sector of leaving edges and a sector of entering edges. Some vertices have only edges that leave the vertex. These are the *peaks*; it is downhill in all directions. Some other vertices have only edges that enter the vertex. These are the *pits*; it is uphill in all directions. Then there are vertices with two entering and two leaving sectors; these are the *saddles*. At a saddle, the downhill stream enters the vertex from two opposite edges and leaves by way of two other opposite edges.

The vertices visited by following the downhill stream in all directions from a peak defines a *hill region*. Defined in this way, hill regions may overlap at their boundaries. We delete these common overlap regions and thus obtain pure hill regions, each belonging to a unique peak.

Each interior edge is shared by two faces. An edge is a regular edge if the downhill stream runs over the edge, which is typical of a sloping region. Some edges are such that the downhill stream runs away from the edge in either direction. These are *ridge edges*. Likewise, some edges are such that the downhill stream runs toward the edge from either direction. These are *rut edges*. Ridge edges can be concatenated to *ridges* and rut edges can be concatenated to *ruts*. At saddle vertices, two ruts and two ridges intersect. These special ruts and ridges play an important role in the topology of the hill regions.

All this is slightly complicated because of the discreteness of the data structure. Otherwise, it is nothing but the familiar structure of geographical landscapes, intuitively understood since ancient times, formalized by Maxwell (1870) and Cayle (1859) in the 19th century.

## References

[bibr1-2041669515615713] Ames A. Jr. (1925a) The illusion of depth from single pictures. Journal of the Optical Society of America 10: 137–148.

[bibr2-2041669515615713] Ames A. Jr. (1925b) Depth in pictorial art. The Art Bulletin 8: 4–24.

[bibr3-2041669515615713] Cayley A. (1859) On contour and slope lines. Philosophical Magazine 18: 264–268.

[bibr4-2041669515615713] Claparède E. (1904) Stereoscopie monoculaire paradoxale. Annales d'Oculistique [Paradoxical monocular stereoscopy]. 465–466.

[bibr5-2041669515615713] Cowan N. (2001) The magical number 4 in short-term memory: A reconsideration of mental storage capacity. Behavioral and Brain Sciences 24: 87–185. 11515286 10.1017/s0140525x01003922

[bibr6-2041669515615713] Ebbinghaus H. (1902) Grundzüge der Psychologie [Fundamentals of psychology], Leipzig, Germany: Veit & Comp.

[bibr7-2041669515615713] Enright J. T. (1991) Paradoxical monocular stereopsis and perspective vergence. In: Ellis S. R. (ed.) Pictorial communication in virtual and real environments, London, England: Taylor & Francis, pp. 567–576.

[bibr8-2041669515615713] Gauss, C. F. (1827). Disquisitiones generales circa superficies curvas [General discourse on curved surfaces]. *Commentationes Societatis Regiae Scientiarum Gottingensis Recentiores, vi*, 99–146.

[bibr9-2041669515615713] Hoffman D. D. Richards W. A. (1984) Parts of recognition. Cognition 18: 65–96. 6543164 10.1016/0010-0277(84)90022-2

[bibr10-2041669515615713] Koenderink J. J. (1990) Solid shape, Cambridge, MA: MIT Press.

[bibr11-2041669515615713] Koenderink J. J. (2014) The all seeing eye? Perception 43: 1–6. 24689127 10.1068/p4301ed

[bibr12-2041669515615713] Koenderink J. J. (2015) PPP. Perception 44: 473–476. 26422897 10.1068/p4405ed

[bibr13-2041669515615713] Koenderink J. J. van Doorn A. J. (1995) Relief: Pictorial and otherwise. Image & Vision Computing 13: 321–334.

[bibr14-2041669515615713] Koenderink J. J. van Doorn A. J. (1998) The structure of relief. Advances in Imaging and Electron Physics 103: 65–150.

[bibr15-2041669515615713] Koenderink J. J. van Doorn A. J. (2003) Pictorial space. In: Hecht H. Schwartz R. Atherton M. (eds) Looking into pictures: An interdisciplinary approach to pictorial space, Cambridge, MA: MIT Press, pp. 239–299.

[bibr16-2041669515615713] Koenderink J. J. van Doorn A. J. (2012) Gauge fields in pictorial space. SIAM Journal on Imaging Sciences 5: 1213–1233.

[bibr17-2041669515615713] Koenderink J. J. van Doorn A. J. Christou C. Lappin J. S. (1996a) Shape constancy in pictorial relief. Perception 25: 155–164. 8733144 10.1068/p250155

[bibr18-2041669515615713] Koenderink J. J. van Doorn A. J. Christou C. Lappin J. S. (1996b) Perturbation study of shading in pictures. Perception 25: 1009–1026. 8983042 10.1068/p251009

[bibr19-2041669515615713] Koenderink J. J. van Doorn A. J. Kappers A. M. L. (1992) Surface perception in pictures. Perception & Psychophysics 52: 487–496. 1437481 10.3758/bf03206710

[bibr20-2041669515615713] Koenderink J. J. van Doorn A. J. Kappers A. M. L. (1994) On so called paradoxical monocular stereoscopy. Perception 23: 583–594. 7800471 10.1068/p230583

[bibr21-2041669515615713] Koenderink J. J. van Doorn A. J. Kappers A. M. L. (1996) Pictorial surface attitude and local depth comparisons. Perception & Psychophysics 58: 163–173. 8838162 10.3758/bf03211873

[bibr22-2041669515615713] Koenderink J. J. van Doorn A. J. Kappers A. M. L. Todd J. T. (2001) Ambiguity and the ‘mental eye’ in pictorial relief. Perception 30: 431–448. 11383191 10.1068/p3030

[bibr23-2041669515615713] Koenderink J. J. van Doorn A. J. Kappers A. M. L. Todd J. T. (2004) Pointing out of the picture. Perception 33: 513–530. 15250658 10.1068/p3454

[bibr24-2041669515615713] Koenderink J. J. van Doorn A. J. Wagemans J. (2011) Depth. i-Perception 2: 541–564. 23145244 10.1068/i0438aap PMC3485797

[bibr25-2041669515615713] Koenderink J. J. van Doorn A. J. Wagemans J. (2012) Picasso in the mind’s eye of the beholder: Three-dimensional filling-in of ambiguous line drawings. Cognition 125: 394–412. 22939735 10.1016/j.cognition.2012.07.019

[bibr27-2041669515615713] Koenderink J. J. Wijntjes M. W. A. van Doorn A. J. (2013) Zograscopic viewing. i-Perception 4: 192–206. 23799196 10.1068/i0585 PMC3690410

[bibr28-2041669515615713] McDermott L. (1996) Self-representation in upper paleolithic female figurines. Current Anthropology 37: 227–275.

[bibr29-2041669515615713] Maxwell J. C. (1870) On hills and dales. Philosophical Magazine 40: 421–427.

[bibr30-2041669515615713] Metzger, W. (1936). *Gesetze des Sehens*. Erstauflage 1936; 2. erweiterte Auflage Verlag Waldemar Kramer, Frankfurt 1953; 3. abermals erweiterte Auflage Verlag Waldemar Kramer, Frankfurt 1975.

[bibr31-2041669515615713] Pollack P. (1955) A note on monocular depth-perception. The American Journal of Psychology 68: 315–318. 14376697

[bibr32-2041669515615713] Pylyshyn Z. W. Storm R. W. (1988) Tracking multiple independent targets: Evidence for a parallel tracking mechanism. Spatial Vision 3: 1–19. 3153671 10.1163/156856888x00122

[bibr33-2041669515615713] Rand W. M. (1971) Objective criteria for the evaluation of clustering methods. Journal of the American Statistical Association 66: 846–850.

[bibr34-2041669515615713] Richards W. A. (1987) Introduction. In: Richards W. A. Ullman S. (eds) Image Understanding 1985–1986, Norwood, NJ: Ablex.

[bibr35-2041669515615713] Rogers B. Graham M. (1982) Similarities between motion parallax and stereopsis in human depth perception. Vision Research 22: 261–270. 7101762 10.1016/0042-6989(82)90126-2

[bibr36-2041669515615713] Rogers L. R. (1969) Sculpture. *In* The appreciation of the arts *(Vol. 2, pp. 51–52)*, London, England: Oxford University Press.

[bibr37-2041669515615713] Schlosberg H. (1941) Stereoscopic depth from single pictures. The American Journal of Psychology 54: 601–605.

[bibr38-2041669515615713] Schwartz A. H. (1971) Stereoscopic perception with single pictures. Optical Spectra 13: 25–27.

[bibr39-2041669515615713] Thompson, K., & Bordwell, D. (2015). *Observations on film art*. Retrieved from htpp://www.davidbordell.net/blog/category/directors-godard/.

[bibr40-2041669515615713] Trick L. M. Pylyshyn Z. W. (1994) Why are small and large numbers enumerated differently? A limited-capacity preattentive stage in vision. Psychological Review 101: 80–102. 8121961 10.1037/0033-295x.101.1.80

[bibr41-2041669515615713] Tyler W. (1974) Depth perception in disparity gratings. Nature 251: 140–142. 4420707 10.1038/251140a0

[bibr42-2041669515615713] van Doorn A. J. Koenderink J. J. Todd J. T. Wagemans J. (2012) Awareness of the light field: The case of deformation. i-Perception 3: 467–480. 23145298 10.1068/i0504 PMC3485835

[bibr43-2041669515615713] van Doorn A. J. Koenderink J. J. Wagemans J. (2011) Rank order scaling of pictorial depth. i-Perception 2: 724–744. 23145256 10.1068/i0432aap PMC3485810

[bibr44-2041669515615713] von Hildebrand, A. (1893). *Das Problem der Form in der bildenden Kunst [The problem of form in the visual arts]*. Heitz, Strassburg.

[bibr45-2041669515615713] Wagemans J. Koenderink J. J. van Doorn A. J. (2013) Pleasures of ambiguity: The case of Piranesi’s Carceri. Art and Perception 1: 121–138.

[bibr46-2041669515615713] Wagemans J. van Doorn A. J. Koenderink J. J. (2010) The shading cue in context. i-Perception 1: 159–178. 23145221 10.1068/i0401 PMC3485766

